# Highlights from the first International *e*cancer Conference on Oncology and Radiotherapy, 6–7 May 2016, Santiago, Chile

**DOI:** 10.3332/ecancer.2016.658

**Published:** 2016-07-22

**Authors:** Leonardo Carmona Reimann, Beatriz Amendola

**Affiliations:** 1 Sociedad Chilena de Cancerologia, Miguel Claro 988, Providencia, Santiago de Chile, 7500000, Chile; 2 Innovative Cancer Institute, 5995 SW 71st St #1B, South Miami, FL 33143, USA

**Keywords:** symposium, cancer, surgery, radiotherapy, eCountouring, radio-surgery, oncology

## Abstract

The first international *e*cancer conference on oncology and radiotherapy was held in Santiago de Chile on 6 and 7 May 2016. It was chaired by Dr Beatriz Amendola, Professor Gordon McVie and Professor Umberto Veronesi. Specialists from Europe and America were invited as guest lecturers.

Over 300 people are attended the conference from all areas of oncology, doctors, physicists, medical technologists, nurses, residents, students, social workers, journalists, pharmaceutical chemists, from all over the country and abroad, including India. The success was achieved as a result of the multidisciplinary approach to the pathologies, which prompted significant interest from the audience.

The ‘eContouring’ course (radio-oncologists) was taken by a large number of participants, the result of great interest in the subject, and the undoubtable merit of the guest instructors.

For the opening ceremony, we were honoured with the presence of Professor Gordon McVie, Founder of *e*cancer, Dr Beatriz Amendola, President of the symposium and Dr Jorge Jimenez, President of Foro Nacional de Cancer Chile, and former Minister of Health.

This conference constitutes *e*cancer’s first conference dedicated exclusively to radiotherapy. Several scientific modules were carried out throughout the conference, with the aim of addressing and updating the community on radiotherapy indications, in cancers that are most prevalent in Latin America. The modules addressed the following topics: lung cancer, breast cancer, gynaecologic cancer, and cancer of the head and neck. The themes were presented in a multidisciplinary manner, in which all of the pillars involved in the treatment of cancer were included, such as surgery, radiotherapy, and medical oncology. We also used the opportunity to discuss, by means of satellite sessions, the current status of radiotherapy in Latin America, and giving participants the opportunity to interact and evaluate new emerging technologies with industry symposia.

An ‘eContouring’ course was conducted, supported by Varian Medical Systems and EduCase, as well as ASTRO (American Society for Radiation Oncology).

This event was held in high regard by the community of oncology professionals in Chile, as it allowed for a multidisciplinary focus such as surgery, radiotherapy, and chemotherapy in the management of patients with cancer. It is important to highlight that in Latin American countries, the studies are carried out in a manner that are relatively isolated from the rest of the community, which is particularly different to what occurs in the rest of the world, specifically Europe, where research is carried out in a coordinated fashion within the different countries, experiences, and findings are shared and no boundaries are present to set back the medical progress in this part of the world. For this reason, contributions, such as the courses, provided by *e*cancer are fundamental to the extinction of these barriers and the emergence of a more team-guided effort to research.

The principal objective of this conference was to demonstrate multidisciplinary cooperation in analysing the different pathologies. With the use of more modern technologies and equipment, considering the pros and cons of each specific one, the individual experiences of the correspondents, their case-by-case interpretation, the potential practical problems, and how to approach them.

In the lung cancer module, Dr Andres Córdova, President of the Radiotherapy Society of Chile, emphasised the importance of tobacco consumption in the appearance of this cancer and called to attention the high prevalence of tobacco consumption in Chile. In addition, Dr Córdova demonstrated the superiority of pre-screening for prevention with low-dosage TAC in relation to radiography of the thorax in high-risk populations, emphasising the importance of prevention. Subsequently, Dr Claudio Suarez, thoracic surgeon of Santa Maria de Santiago Clinic, discussed the different types of surgical interventions, their advantages and disadvantages, as well as how a good programme for early detection can greatly improve the statistics for survival.

Dr Beatriz Amendola of the ‘Innovative Cancer Institute’ Miami, EEUU, presented lectures in relation to radiosurgery in general and the management of metastasis in lung cancer. Dr Amendola emphasised the increase in the incidence and negative results of treatment in cases of late detection. It was demonstrated how stereotactic body radiation therapy (SBRT) has been gaining prevalence, specifically in elderly patients or those patients presenting comorbidities that present an impediment to surgical intervention. With regards to the treatment of metastasis, the importance of SBRT in vertebral, hepatic, and cerebral metastasis, and the associated subdivisions, was demonstrated.

Dr Lus E Raez, of the Memorial Cancer Institute, discussed the systematic management of Lung Cancer, with particular focus on personalized therapies and the positive outcomes seen with the use of TKI, with ALK and ROS-1 inhibitors in selected patients. Dr Raez also discussed Immunotherapy and its results in cerebral and hepatic metastasis.

Dr Marco Amendola of the ‘Innovative Cancer Institute’, discussed imaging techniques in lung cancer, referring to the staging, the follow-up and restaging, highlighting the importance of imaging for the organisation of radiotherapy, using the different techniques, particularly the use of positron emission tomography-computed tomography (PET-CT).

In the module discussing breast cancer and global radiotherapy, Dr Maria Laplana of the Catalan Institute of Oncology presented a conference on IORT (intraoperative radiotherapy), SBRT (stereotactic body radiation therapy), and HDR-IGBT (high-dose rate image-guided brachytherapy), comparing the different techniques and discussing the statistics regarding them. Subsequently, Professor Gordon McVie, of King’s College London, Founder of *e*cancer, presented the following: Life After Protons, also referring to the successful evolution of *e*cancer since its foundation in 2007. In regards to protons, Professor McVie explained the characteristics and differences from photons, as well as their application in adults and children. Images of isodose were demonstrated throughout different locations, such as liver, head and neck, and breast.

Dr Herbert Cardenas, radio-oncologist at the National Institute of Neoplastic Diseases (INEN) Peru, demonstrated his experience with V-MAT (volumetric modulated arc therapy), comparing this approach with fixed IMRT in different locations and its individual advantages.

Dr Ana Maria Ciudad, of the Arturo Lopez Perez Foundation, Santiago de Chile, presented the Uterine Cancer module. Dr Ciudad discussed the integral management of cervical and uterine cancer in Chile, providing mortality rates and their evolution in time, as well as demonstrating a national research study, presenting figures from different stages and their treatment outcomes and results. Dr Clemente Arab, gynaecologist of the Arturo Lopez Perez Foundation and the hospital of Tisne de Santiago, discussed the topic of surgery in gynaecological cancers, indications with parametrectomy, types of surgery in low-risk tumours, the use of sentinel ganglions, and the use of radical abdominal trachelectomy.

Dr Arno Mundt, radio-oncologist of Moffit Center, Florida, talked about radiotherapy in tumours of cervical cancer and the combination with surgery and/or chemotherapy, neoadjuvant chemotherapy with varied schemes, radio-chemotherapy and novel drugs. Dr Mundt also discussed the novel uses of radiotherapy in cancer of the vulva, endometrial cancer, and selected patients with ovarian cancer.

In the international presentation, Dr Bernardo Vizcarra of Oncosalud Peru referred to radiotherapy and its evolution in time, as well as its applications in Peru, specifically in lesions of the central nervous system (CNS).

The session presented by Dr Hugo Marsiglia, of the Arturo Lopez Perez Foundation, Santiago de Chile, discussed the 2016 consensus in radiotherapy for breast cancer. Dr Marsiglia demonstrated research studies carried out in patients were or were not treated with radiotherapy, in an elderly population of patients with breast cancer, and the clear advantage of treating with radiotherapy, the use of IOERT in young patients, various subdivisions and treatment of ganglionic areas.

In the subsequent module, addressing cancer of the head and neck, Dr Adela Poitevin of Medica Sur, Mexico, discussed the general principles of the management and diagnosis of this type of pathology, as well as its prevalence in Latin America. Dr Poitevin demonstrated rates, risk factors, and treatment concepts.

Dr Beatriz Amendola analysed radio-surgery (SBRT) in recurrent head and neck cancer, re-irradiation, as well as different techniques and dosage. Dr Amendola demonstrated different cases with their respective isodose curves, in different localisations and their encouraging results.

Dr Gustavo Sarria, radio-oncologist of Oncosalud, Peru, discussed advanced techniques in regards to treatment of cancer of the head and neck, the results observed with IORT, brachytherapy, the evolution of different techniques of external radiotherapy and the comparison of V-MAT and fixed gantry IMRT.

The following session was in the hands of Dr Beatriz Amendola. Dr Amendola discussed radiosurgery in the base of the skull and cervical vertebrae, particularly meningioma and vertebral metastasis, highlighting its promising results. Practical recommendations were given in regards to the delineation of volumes and potential risks of complications. Following this conference, Dr Luis E. Raz presented a discussion on the growth and evolution of the systemic management of head and neck cancer, particularly the use of immunotherapy, the importance of HPV, chemotherapy for induction and alternative schemes.

The conference presented by Dr Arturo Madrid, surgeon of the Head and neck in the German Clinic of Santiago de Chile, discussed robotics, highlighting its advantages in lesions of the amygdala, the learning curve and potential use in other locations. Following this presentation, Dr Eugenio Vines, of the Service of Radiotherapy, Catholic University of Santiago, discussed re-irradiation in head and neck cancer. Dr Vines demonstrated cases of recurrent disease and the incidence of secondary primary malignancies with different re-irradiation techniques and their results.

Dr Claudio Sole, radio-oncologist of the IRAM Oncology Clinic of Santiago, presented a session discussing the medical advances and novel trends in the treatment of prostate cancer. Dr Sole elaborated on different treatments for specific risk factors, dose escalation and varied fragmentation.

Dr Sebastián Solé, also from the IRAM Oncology Clinic of Santiago, discussed novel strategies for the treatment of locally advanced rectal cancer, comparing specifically conventional radiotherapy versus shortened radiotherapy, and the importance of time periods in regards to treatment.

To bring the conference to an end, Dr Arno Mundt discussed the medical advances in radiotherapy and its application to gynaecological cancer. Dr Mundt compared the varied techniques by means of their use, advantages, disadvantages, results and their complications.

During the eContouring course, which attracted a large number of participants, themes regarding the use of eContouring in lung, cervical, uterine, endometrial, prostate, head, and neck cancer were analysed. The guest instructors Dr Adela Poitevin, Dr Arno Mundt, Dr Marco Amendola, and dosimetrist Alejandro Iglesias, provided a brief introduction, a review of the anatomy and volumes to embark upon, as well as potential constraints. It is worth noting that prior to this course, participants were tasked with several assignments, to be carried out in their homes. It was a complete success.

In addition, the presence of satellite symposia allowed the participants to experience the latest projects and technologies of the industry: Elekta, Reich, Varian and Siemens were all participating.

## Conclusion

The meeting was valuable as the topics were very interesting and of current importance. Similar meetings are expected to be replicated in other Latin American countries.

